# Numerical and experimental investigation of multi-species bacterial co-aggregation

**DOI:** 10.1038/s41598-023-38806-2

**Published:** 2023-07-22

**Authors:** Meisam Soleimani, Szymon P. Szafranski, Taoran Qu, Rumjhum Mukherjee, Meike Stiesch, Peter Wriggers, Philipp Junker

**Affiliations:** 1 Institute of Continuum Mechanics (IKM), Leibniz Universität Hannover, Hannover, Germany; 2 Medical School Hannover (MHH), Hannover, Germany

**Keywords:** Biofilms, Computational models

## Abstract

This paper deals with the mathematical modeling of bacterial co-aggregation and its numerical implementation in a FEM framework. Since the concept of co-aggregation refers to the physical binding between cells of different microbial species, a system composed of two species is considered in the modeling framework. The extension of the model to an arbitrary number of species is straightforward. In addition to two-species (multi-species growth) dynamics, the transport of a nutritional substance and the extent of co-aggregation are introduced into the model as the third and fourth primary variables. A phase-field modeling approach is employed to describe the co-aggregation between the two species. The mathematical model is three-dimensional and fully based on the continuum description of the problem without any need for discrete agents which are the key elements of the individual-based modeling approach. It is shown that the use of a phase-field-based model is equivalent to a particular form of classical diffusion-reaction systems. Unlike the so-called mixture models, the evolution of each component of the multi-species system is captured thanks to the inherent capability of phase-field modeling in treating systems consisting of distinct multi-phases. The details of numerical implementation in a FEM framework are also presented. Indeed, a new multi-field user element is developed and implemented in ANSYS for this multiphysics problem. Predictions of the model are compared with the experimental observations. By that, the versatility and applicability of the model and the numerical tool are well established.

## Introduction

Most bacteria inhabiting the human mouth exhibit physical cell-to-cell recognition and binding of organisms coming from different representative taxa, see^1,2,3^ and^4^. This phenomenon is called co-aggregation if it occurs in suspension and it is called co-adhesion if it involves immobilized microorganisms. These microorganisms co-adhere to each other using high number of attachment points and special physical interactions, thus staying in close proximity to each other. Physical interactions are evolutionarily conserved in oral microorganisms because they protect microbial cells from being washed away from the oral surface by the constant flow of saliva and consequently being swallowed by the host^5^. Primary colonizers can bind to oral surfaces, while secondary colonizers must rely on other organisms to be recruited into the sessile community.

Interspecies cell-cell binding occurs rapidly and usually in a very specific manner as proved by co-aggregation assays performed for multiple strains representing a broad range of oral species^2^. Testing inhibitory substances/conditions and/or genetic studies revealed that most often co-aggregation is mediated by a carbohydrate-binding protein called lectin on one cell type that interacts with a complementary carbohydrate receptor on the other cell type. Other surface components, like outer membrane proteins, hemagglutinins and sialic acid-containing receptors, can also be involved in a co-aggregation process^6^. Microorganisms take advantage of proximal location facilitated by physical contact, to co-evolve and develop bidirectional beneficial relationships. Close distance between interacting cells supports cross-feeding, cross-protection, gene transfer, and cell-cell signaling, see^3,7,8^ and^9^. Hence, co-aggregation plays a pivotal role in oral biofilm development by governing the sequential recruitment of different species, their spatial distribution and ecology. Recently, a cell-by-cell succession model was extended to encompass direct adhesion of co-aggregates, see^10^. Interestingly, *in vitro* biofilm initiated by aggregates expanded more rapidly compared to biofilms developing from single cells. Dental plaque is the most extensively investigated biofilm in this regard. It is known that more than 500 species contribute to the formation of human dental plaque. Streptococci and actinomyces are the major and primary colonizers of the tooth surface^11^.

From a clinical perspective, the development and uncontrolled expansion of oral biofilms can lead to diverse pathologies, primarily local but potentially with subsequent systemic complications. Oral biofilms are responsible for the most prevalent infectious disease worldwide, dental caries and periodontal disease, and the most common implant-associated complications, peri-implant diseases. Odontogenic complications include pathologies at diverse anatomic locations including the cardiovascular system, gut, respiratory tract, reproductive tract, or even brain. Physical contact between biofilm members contributes to biofilm structure that facilitates higher resistance to immune response and antimicrobial treatment. On the one hand, co-aggregation can directly contribute to virulence in a process of synergistic invasion^12^ or invader recruitment^13^. On the second hand, receptors involved in co-aggregation can be used to target pathogens, with the use of antimicrobials and probiotics^14^. Given the critical role of cell-cell contact in biofilm development and maturation, it can be speculated that a better understanding of this process could result in improved prophylaxis and anti-biofilm therapy. Yet, the holistic approach to oral microbial ecology is challenged by the high diversity and complexity of microbial communities. High-resolution numerical simulations can help to overcome current methodological limitations and improve a system-level understanding of oral biofilms.

The mathematical modeling and numerical simulation of bacterial colonies (biofilms) have drawn a great deal of attention among researchers over the last two decades. In particular, the spatiotemporal evolution of bacterial aggregates is of great interest in the computational biomechanics community. A common and favorable modeling scheme is Individual-based modeling (IBM) which is very flexible in terms of accommodating complex interactions between the discrete agents as well as sophisticated biological, chemical, and physical processes associated with a single cell such as microbial cell division, decay, motility, etc. Moreover, this approach is readily scalable in the sense that an extremely large number of microbial populations can be handled using parallel solvers and domain decomposition due to the explicit nature of their time integration scheme. As the outcome of such endeavors, one can name several open-source computer codes such as iDynoMiCS^15^ and Simbiotics^16^, NUFEB^17^ and also MIRODIM^18,19^ which are capable of simulating the development of microorganism in a multi-dimensional and multi-species fashion. IBMs are normally of hybrid type meaning that besides the bacterial species, which are treated as discrete agents, the nutrient availability is described using a continuum approach. Along with IBMs, fully continuum-based modeling of biofilms is also mathematical modeling and the numerical simulation of the multi-species biofilms have not yet been sufficiently considered in the literature. Although most species that constitute dental plaque are known individually, their collective behavior and group interactions are still under investigation. In order to understand the spatiotemporal structure of such a bacterial community one has to identify and understand the interaction, partnership and communication among the members of this community. In particular, the mathematical modeling of such phenomena is really sparse in the literature.

When it comes to mathematical modeling, the growth of biofilms can be viewed from two different perspectives. In the first approach, the growth is described as volume expansion and consequently, a “mechanical movement” of the boundary of the growing matter. This “explicit” movement of the biofilm can be captured, in the simplest fashion, using a potential flow which is isotropic, see for example^20^ and^21^. Alternatively, in a more complex framework, one can use anisotropic kinematics that governs the deformation of the biofilm as a deformable viscoelastic solid instead of a fluid, see^22^ and^23^. This notion of growth has also been employed to model various growth phenomena in soft tissues other than bacterial aggregates. Examples are tumor growth^24^, and aneurysm as a result of abnormal arterial growth^25^. In the second approach, the growth of biofilm can be characterized using the “propagation” of a phase-field variable. A classical example is the classical diffusion-reaction equation in which the propagation of the wavefront can be interpreted as the growth of the colony of species, see^26,27,28–30^. Another example is Fisher’s equation^31^ which is, indeed, a transient diffusion-reaction model for predicting population dynamics and pattern formation. One can generalize this framework by using a set of diffusion-reaction equations in order to account for different reciprocal interactions, such as symbiosis or competition, between multiple species. For instance, the diffusional Lotka-Volterra system^32^ is associated with a set of PDEs capable of describing the interaction between two species which are, in fact, prey and predator.

In this work, we use the second approach in order to mathematically describe a system composed of two different bacterial species which not only grow but also co-aggregate in the presence of each other. Moreover, it is shown that this approach is exactly equivalent to employing a so-called “phase-field” model for capturing the behavior of such biological systems. The mathematical model is implemented using a fully implicit and monolithic scheme within a FEM framework. A multi-physics user element is developed from scratch to be invoked by the ANSYS solver. Several numerical examples are presented to show the robustness of the proposed numerical tool in handling multi-species biological systems.

## Mathematical modeling of the co-aggregation of two bacterial species

From a biological point of view, co-aggregation is a crucial process in the formation of multi-component biofilms. Genetically distinct bacteria adhere to each other, ’co-aggregate’, and can replicate faster, see^33^. Due to co-aggregation, the metabolism of the species is altered compared to the monocultures, see^3^. In order to mathematically describe the development of biofilm under the influence of co-aggregation, the basic assumptions are:

- Two species of bacteria are taken into consideration. One can readily extend the model to multiple species without any restriction.
- For the sake of simplicity, only a single nutrient is assumed to nourish both species. The nutrient is, indeed, dissolved in the fluid surrounding the biofilm. One can extend the model to multiple nutrients.
- Since the fluid flow is not modeled explicitly, the transport of the nutrient is governed by the diffusion mechanism. This is in line with a very small flow velocitythat is either induced by the biofilm growth (in the order of a few microns per day) or governed by quasi-hydrostatic conditions. As a result, the advection mechanism for the transport is neglected. Such a situation can occur in specific anatomic locations, e.g., deep pockets around implants or teeth, where salivary flow is strongly reduced.a very small Peclet number (Pe) allows such simplification regarding the transport mechanism. One can estimate that $\text {Pe}=\frac{\text {Advection}\,\text {rate}}{\text {Diffusion}\, \text {rate}}=\frac{Lu}{D}\rightarrow 0$ in which $L, \ u$ and *D* refer to the characteristic length, characteristic fluid velocity, and nutrient diffusivity, respectively.
- The generation of so-called byproducts, as a result of the metabolic activities of the bacteria, is not modeled explicitly. It is known that metabolic products, such as lactic acid, can have either a negative or positive influence on the growth of different components in multi-species systems^34^.
- The model of the co-aggregation is mathematically based on the “proximity” of species. Irrespective of the underlying biological mechanism, the model assumes that bacterial growth is boosted if particular species are “sufficiently close” to each other. In this sense, co-aggregation can be perceived as cooperation.
- It is postulated that the growth of a biofilm colony is in the direction of the maximum nutrient gradient. It will be clarified more in the mathematical description of the proposed phase-field model.
- The initial seeds of species are randomly generated in the geometrical model. The model does not capture the very early colonization of the surface by the planktonic bacteria. Such a process needs to be modeled using agent-based methods with regard to the length scale of bacteria and the surface roughness, see^35^.

**Figure 1:**
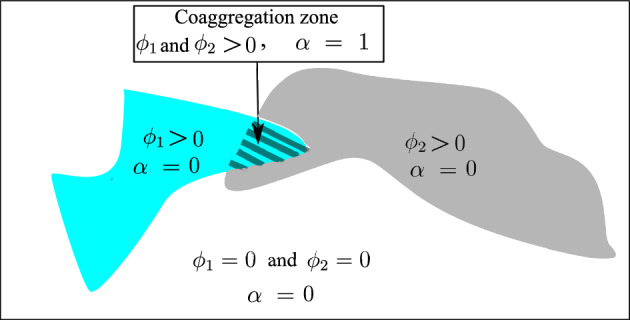
Phase-field approach for a multi-species system, $\phi _1$ and $\phi _2$ represent species 1 and species 2 while $\alpha$ refers to the congregation state.

The mathematical model is based on these assumptions. For the sake of simplicity, the schematic view of the computational domain is considered to be two-dimensional. The variables $\phi _1$ and $\phi _2$ are taken to represent species 1 and 2 respectively. These two variables can be perceived as the density of bacteria. If they are equal to zero at a certain location, it implies the absence of that species. Finally, the sharp interface between zero-valued and one-valued regions characterizes the boundary of the species. Figure 1 depicts the regions where $\phi _1$ and $\phi _2$ change spatially.

One can see that both species may coexist (both $\phi _1$ and $\phi _2$ amount to 1) in certain regions. Such regions are exactly the aggregation zones. The aggregation is captured using a new variable called $\alpha$. This variable can be evaluated by applying a Boolean operation of “AND” type on $\phi _1$ and $\phi _2$. This variable is treated like a phase-field variable. It means that $\alpha =1$ corresponds to the regions in which both $\phi _1$ and $\phi _2$ are present and consequently the co-aggregation can take place.

Finally, the concentration of the nutrient is denoted by the variable *c* that obeys a diffusion-reaction equation. It is obvious that the regions occupied by one of the species, namely $\phi _1$ and/or $\phi _2$ equal to 1, play the role of sink term (reaction) in the diffusion-reaction equation. Furthermore, the values of nutrients on the boundaries can be prescribed/controlled in the context of boundary conditions.

### Phase-field type equations for species and co-aggregation

As mentioned before, the variable $\alpha$ is captured using a phase-field equation. The well-known Allen–Cahn^36^ type of phase-field equation can be written as

1 $$\begin{aligned} \underbrace{ {\dot{\square }}}_{\begin{array}{c} \text {Phase evolution} \\ \text {in dimensionless time} \end{array}}=\underbrace{-f'(\square )}_{\begin{array}{c} \text {Bulk contribution} \\ \text {} \end{array}}+\underbrace{\varepsilon ^2 \nabla ^2 \square }_{\begin{array}{c} \text {Sharp interface} \\ \text {contribution} \end{array}}+\underbrace{S(\square ,c)}_{\begin{array}{c} \text {Driver (source)} \\ \text {of phase-field} \end{array}}, \end{aligned}$$

in which the generic variable $\square$ can be replaced by either $\phi _1$, $\phi _2$ or $\alpha$. The dot over $\square$ in the first term signifies the time derivative. The parameter $\varepsilon$ regularizes the sharp change of $\phi$ between the two phases. $f'(\square )$ is a short notation for $\frac{\partial f(\square )}{\partial \square }$. The function $f(\square$) is a so-called double-well potential and characterizes the barrier that has to be overcome in order to have a phase transformation (here, phase transformation means a region that used to be free of species, namely $\square =0$, is colonized by that species, namely $\square =1$). The description of each term in the equation is self-explanatory and standard in any application of phase-field modeling.

**Figure 2:**
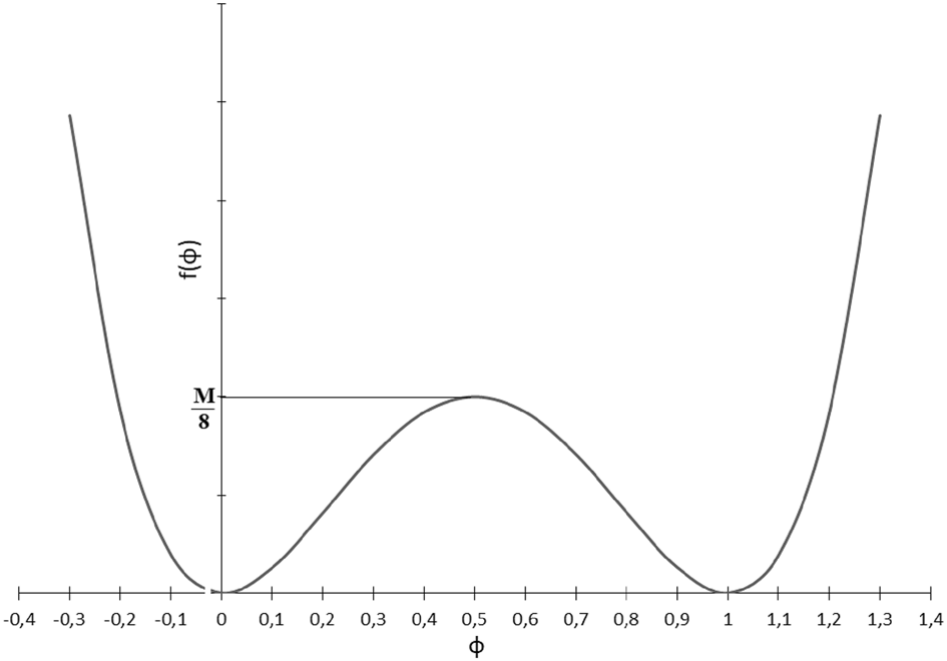
Double well potential $f(\phi )$.

A common choice for the double well potential is

2 $$\begin{aligned} f(\square )=2 M \square ^2(1-\square )^2, \end{aligned}$$

in which *M* is proportional to the local maximum of the function at $\phi =\frac{1}{2}$ between the two wells (minima) at $\square =1$ and $\square =0$, see Fig. 2. By degenerating the problem to 1D one can better understand the idea behind the phase-field approach. Neglecting the time-dependent as well as the source term, in 1D, one can realize that the so-called wavefront function $\phi =\frac{1}{2}(1-\text {Tanh}(\frac{x}{l})$ satisfies Eq. (1). Here $l=\sqrt{\frac{\varepsilon ^2}{M}}$ characterizes clearly the “interface width”. It captures a smooth but sharp transition between the two phases. In a similar fashion, one can show that the “interface energy” is proportional to $\sqrt{\varepsilon ^2 M}$. In other words, it represents the resistance against phase change. If the driving force (the last term in Eq. 1) overcomes this threshold, the phase change takes place. Actually, $\varepsilon$ and *M* are characteristic parameters that govern the underlying physical process pertaining to the phase-field equation. The readers are referred to^37^ for more elaboration on the structure of the phase-field equation.

As stated before, the co-aggregation ($\alpha$) can be captured using the phase-field equation. It is noteworthy to mention that this type of phase-field modeling is, in fact, a transient diffusion-reaction equation. To restore the classical diffusion-reaction equation one can eliminate the term concerning double well potential (by setting $M=0$). In fact, the evolution of the two species’ density is governed by the classical diffusion-reaction equation. To do so, one can substitute $\square$ for $\phi _1$ or $\phi _2$ and set $M=0$.

The crucial part of the phase-field equation is the definition of the source terms for each field. The source terms corresponding to the four variables $\phi _1,\phi _2,\alpha ,c$ are denoted by $S_1(\phi _1,\alpha , c)$, $S_2(\phi _2,\alpha , c)$, $S_{\alpha }(\phi _1,\phi _2,\alpha , c)$ and $S_c(\phi _1,\phi _2, c)$, respectively. That gives rise to the evolution of the system. Its mathematical representation needs to be physically meaningful. As stated in the assumptions, the “physically meaningful rationale” is that the expansion of bacterial colonies is preferred in the direction of maximum nutrient change, namely nutrient gradient. Nutrient metabolism provides energy and building blocks for all cell replications. Consequently, in locations where access to nutrients is hindered, replication is slower or stops completely. In the following, appropriate source terms are considered.

The function $S_1(\phi _1,\alpha ,c)$ can be proposed as

3 $$\begin{aligned} S_1(\phi _1,\alpha ,c)=R_{s1} H(\phi _1-\phi _{{\text {cri}}}) (1+ \tau \alpha ) \phi _1 c, \end{aligned}$$

where $R_{s1}$ is a parameter that controls the magnitude of the source term and $\tau$ characterizes the degree of growth promotion due to coaggregation. It is hypothesized that the growth (bacterial replication) is boosted if $\alpha$ is evolved (co-aggregation takes place). As long as different species have positive effects on each others’ fitness/growth, closer proximity introduced by co-aggregation can facilitate stronger synergy. The function $H(\phi _1-\phi _{\text {{cri}}})$ is the Heaviside step function with a jump at critical value $\phi _{\text {cri}}$. A general definition of $H(\cdot -\cdot _{\text {cri}})$ with a jump at $\cdot _{\text {cri}}$ is

4 $$ H( \cdot  -  \cdot _{{{\text{cri}}}} ) = \left\{ {\begin{array}{*{20}c}    1 & {{\text{If }} \cdot  \le  \cdot _{{{\text{cri}}}} }  \\    0 & {{\text{If }} \cdot  >  \cdot _{{{\text{cri}}}} }  \\   \end{array} } \right.. $$

One can interpret the parameter $\phi _{\text {cri}}$ as the minimum level of bacterial density that prevents the bacterial colonies from vanishing, in the long run. The source term, which can be interpreted as bacterial replication, is proportional to bacterial density and nutrient availability. The source term for species 2 is similar to species 1

5 $$\begin{aligned} S_2(\phi _2,\alpha ,c)=R_{s2} H(\phi _2-\phi _{\text {cri}})(1+ \tau \alpha ) \phi _2 c. \end{aligned}$$

The source term for the co-aggregation variable $\alpha$ can be written as

6 $$\begin{aligned} S_{\alpha }(\phi _1,\phi _2,\alpha ,c)=R_{\alpha } H(\phi _1-\phi _{\text {cri}})H(\phi _2-\phi _{\text {cri}}) \frac{\nabla \alpha \cdot \nabla c}{|\nabla c|}, \end{aligned}$$

in which the term $\frac{\nabla c}{|\nabla c|}$ is a unit vector pointing to the direction of maximum change in the nutrient. This is, indeed, the preferred direction for the expansion of colonies. Taking a closer look at the structure of $S_{\alpha }(\phi _1,\phi _2,\alpha ,c)$, one can notice that it serves as an advection term in the form of ${{v}} \cdot \nabla \alpha _1$ and ${{v}}=R_{\alpha } \frac{\nabla c}{|\nabla c|}$ being the advection velocity. It means that the boundary of the bacterial colonies at the co-aggregation zone is advected in the direction of *v*.

For the nutrient *c*, the source term should be negative, because the nutrient is consumed.

7 $$\begin{aligned} S_c(\phi _1,\phi _2,c)=-R_c ( \phi _1+ \phi _2)c, \end{aligned}$$

in which the parameter $R_c$ is the consumption parameter. Obviously, the transport of the nutrient is governed by the standard and classical time-independent diffusion-reaction equation ($M=0$). For the nutrient, the parameter $\varepsilon ^2$ in Eq. (1) is, in fact, the nutrient diffusivity. To make a distinction, we replace $\varepsilon ^2$ with $D_n$ and $D_b$ in the case of nutrient and bacterial species, respectively.

## Method

All methods were performed in accordance with the relevant guidelines and regulations.

### Numerical implementation using FEM

Since the adopted numerical method is based on the standard Galerkin FEM, one needs to convert the differential equations into their respective weak forms. One can express the weak form of four governing equations for the existing four unknowns ($\phi _1$, $\phi _2$, $\alpha$ and *c*) as follows:

8 $$\begin{aligned}&\int _{\mathscr {B}} \left[ (D_b\nabla \cdot \nabla \phi _1) \delta \phi _1 - S_1(\phi _1,\alpha ,c)\delta \phi _1 +{\dot{\phi }}_1 \ \delta \phi _1 \right] \ dv =0, \end{aligned}$$

9 $$\begin{aligned}&\int _{\mathscr {B}}\left[ (D_b\nabla \cdot \nabla \phi _2) \delta \phi _2 - S_2(\phi _2,\alpha ,c)\delta \phi _2 +{\dot{\phi }}_2 \ \delta \phi _2 \right] \ dv =0, \end{aligned}$$

10 $$\begin{aligned}&\int _{\mathscr {B}}\left[ (\varepsilon ^2\nabla \cdot \nabla \alpha ) \delta \alpha + f'(\alpha )\delta \alpha -S_{\alpha }(\phi _1,\phi _2,\alpha ,c)\delta \alpha +{\dot{\alpha }} \ \delta \alpha \right] \ dv =0, \end{aligned}$$

11 $$\begin{aligned}&\int _{\mathscr {B}} \left[ D_n\nabla \cdot ( \nabla c ) \delta c+S_c(\phi _1,\phi _2,c) \delta c+\dot{c} \ \delta c\right] \ dv =0. \end{aligned}$$

One should note that variation of the variables ($\delta \phi _1,\delta \phi _2,\delta \alpha ,\delta c$) serve as the test function in the context of variational approach. Furthermore, $(\cdot )$ acting between the variable gradients is indeed the inner product. In line with standard FEM, the objective is to find the field variables with so-called $C_0 \ \text {continuity}$. Upon applying integration by parts to the integrals in Eqs. (8)–(11), the boundary terms defined on the boundary ${\partial {\mathscr {B}}}$ emerge. Furthermore, a backward (implicit) Euler scheme is used for the time discretization which replaces the time derivatives. This yields

12 $$\begin{aligned}&\int _{\mathscr {B}}\left[ D_b\nabla \phi _1 \cdot \nabla \delta \phi _1 +\frac{\phi _1-\phi _1^{n-1}}{\Delta t} \delta \phi _1 -S_1(\phi _1,\alpha ,c)\delta \phi _1 \right] \ dv=\int _{\partial \mathscr {B}} D_b\nabla \phi _1 \cdot {{N}} \ \delta \phi _1\ ds, \end{aligned}$$

13 $$\begin{aligned}&\int _{\mathscr {B}}\left[ D_b\nabla \phi _2 \cdot \nabla \delta \phi _2 +\frac{\phi _2-\phi _2^{n-1}}{\Delta t} \delta \phi _2 -S_2(\phi _2,\alpha ,c)\delta \phi _2 \right] \ dv=\int _{\partial \mathscr {B}} D_b\nabla \phi _2 \cdot {{N}} \ \delta \phi _2\ ds, \end{aligned}$$

14 $$\begin{aligned}&\int _{\mathscr {B}}\left[ \varepsilon ^2\nabla \alpha \cdot \nabla \delta \alpha + f'(\alpha )\delta \alpha +\frac{\alpha -\alpha ^{n-1}}{\Delta t} \delta \alpha -S_{\alpha }(\phi _1,\phi _2,\alpha ,c)\delta \alpha \right] \ dv=\int _{\partial \mathscr {B}} \varepsilon ^2\nabla \alpha \cdot {{N}} \ \delta \alpha \ ds, \end{aligned}$$

15 $$\begin{aligned}&\int _{\mathscr {B}} \left[ D_n \nabla c \cdot \delta \nabla c\ +S_c(\phi _1,\phi _2,c) \delta c +\frac{c-c^{n-1}}{\Delta t} \delta c\right] \ dv =\int _{\partial \mathscr {B}} D_n \nabla c\cdot {{N}} \ \delta c\ ds, \end{aligned}$$

where *N* is the normal vector of the boundary surface. It is an acceptable assumption that the boundary flux term for the phase-field is assumed to be zero. Moreover, $D \nabla c\cdot {{N}}$ refers to the nutrient flux at the boundary. The superscript ($n-1$), which appears due to time discretization, refers to the values of variables at the previous time step.

The implementation of the multi-field (species 1, species 2, co-aggregation, and nutrient concentration) three-dimensional problem at hand was carried out using AceGen, see^38^, which is based on automatic differentiation (hybrid symbolic/numeric differentiation). Once the weak forms are linearized, the stiffness matrix is extracted and tailored to a user element in the FORTRAN language that can be implemented in any FEM code using standard discretization schemes. The numerical test cases are performed on two-dimensional models for the sake of computational efficiency. Due to the nature of phase-field equations, one needs to use sufficiently fine mesh. Even in two-dimensional examples, a mesh size of 0.5 $$\mu \text {m}$$ leads to approximately $$2\times 10^4$$ nodes which means around $$8\times 10^4$$ degrees of freedom (DoF) in the final model. One can easily understand that for a three-dimensional model with the same mesh size, the total number of DoFs exceeds $$ 10^7$$ and is computationally prohibitive.

Each node of the FEM mesh has four degrees of freedom all of which are scalar-valued. The first and second ones are dedicated to $\phi _1$ and $\phi _2$ representing the density of species 1 and species 2, respectively. The third is $\alpha$ which captures the co-aggregation degree of the two species. The fourth one is allocated to the concentration field *c*. The solution of the global system yields the updated value of the field variables, with the assumption that the previous values ($\phi _1^{n-1}$,$\phi _2^{n-1}$,$\alpha ^{n-1}$ and $c^{n-1}$) are known.

In the next section, several numerical examples are presented to see the applicability of the numerical tool. The post-processing and visualization of the results were conducted in Paraview.

### Numerical and experimental test cases

**Figure 3:**
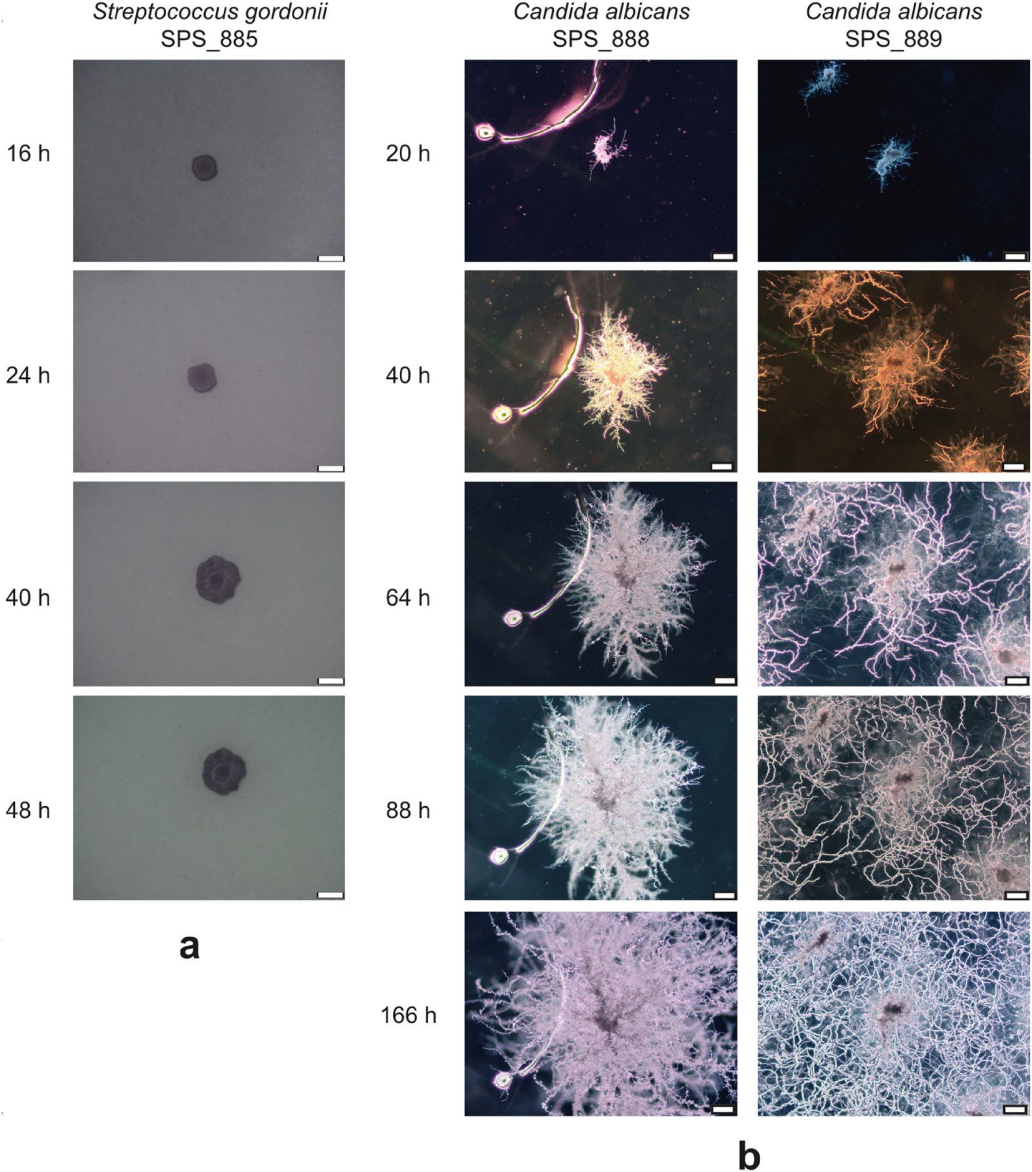
Two different patterns for development of biofilms. (**a**) Phase-contrast micrographs of colony morphology for Streptococcus Gordonii strain SPS$\_$885. The colony is generally circular and compact. A slightly undulate colony margin was observed at later time points. (**b**) Colony morphologies of Candida albicans strains SPS$\_$888 and SPS$\_$889. Colonies are rhizoid (branched and dendritic pattern) with irregular cores. Merging of colonies was observed for strain SPS$\_$889 at later time points. Scale bars, 100 $\mu m$.

For the numerical simulations, all geometrical data as well as material parameters are summarized in Table 1. the temporal unit (denoted by “Time” in the table) is nondimensionalized using growth characteristic time scale which is in the order of a few hours, see Fig. 3. It means that, from a numerical point of view, “Time” varies between 0 and 1.

**Table 1:** Model parameters.

Description	Parameter	Value	unit
Nutrient diffusivity	$D_n$	$10^{-6}$	$\mu \text {m}^2\ \text {Time}^{-1}$
Bacterial density threshold	$\phi _{\text {cri}}$	0.1	$\mu \text {g}\ \mu \text {m}^{-3}$
Consumption rate	$$R_c $$	50	$$ \mu \text {m}^3\ \mu \text {g}^{-1} \text {Time}^{-1}$$
Co-aggregation coef.	$R_{\alpha }$	15	$\mu \text {m}\ \text {Time}^{-1}$
Co-aggregation coef.	$\tau$	1	–
First species growth coef.	$R_{s1}$	500	$\mu \text {m}^3\ \mu \text {g}^{-1} \text {Time}^{-1}$
Second species growth coef.	$R_{s2}$	500	$\mu \text {m}^3\ \mu \text {g}^{-1} \text {Time}^{-1}$
Phase-field coefficient for $\alpha$	*M*	$10^{-4}$	$\text {Time}^{-1}$
Phase-field coefficient for $\phi _1,\phi _2 \ \text {and}\ c$	*M*	0	$\text {Time}^{-1}$
Phase-field coefficient (diffusivity)	$\varepsilon ^2 (D_b)$	$10^{-8}$	$\mu \text {m}^2\ \text {Time}^{-1}$
2D Computational domain size		$100 \times 100$	$\mu \text {m}$
Mesh size		0.5	$\mu \text {m}$

### Approval for human/animal experiments

This article does not contain any studies with animals performed by any of the authors. The humans were involved in terms of collecting dental peri-implant bacterial samples. The collection of clinical samples was authorized by the ethics committee of Hannover Medical School (No. 9477) in accordance with the principle of anonymity. Furthermore, informed consent was obtained from all the participants.

### Test case 1: single colony, single species

In the first example, different patterns of growth are studied. A single tiny colony is assumed to be located at the center of the domain. The initial nutrient distribution is uniform and constant. The numerical investigation shows that the ratio between the nutrient and biofilm diffusivity ($r_=\frac{D_n}{D_b}$) plays an important role in the morphology of the grown biofilm. Hereafter, two distinct cases are investigated. It is assumed that the entire domain is enriched by a uniform initial nutrient. There is no external provision of the nutrient. Hence, the nutrient can only be consumed. To validate the reliability of the mathematical model, experimental observations are also conducted.

#### Experimental setting: bacterial cells and culture conditions

The experimental results are depicted in Fig. 3. The bacterial strains used were *Eikenella corrodens* strain SPS$\_$010, Streptococcus Gordonii strains SPS$\_$017 and SPS$\_$886 (both isolated from the same case of severe peri-implantitis), as *Candida* albicans strains SPS$\_$888 (= ATCC 10231, DSM 1386, NIH 3147) and SPS$\_$889 (= ATCC 90028, DSM 11225, NCCLS 11), as well as oral fungal isolate strain SPS$\_$887 from human peri-implantitis. Bacteria were cultured on Fastidious Anaerobe Agar (LabM) supplemented with 5% sheep blood. Fungal strains were cultured on Czapek Dox Agar (Sigma-Aldrich). Bacterial colonies were used to inoculate Todd-Hewitt Broth supplemented with 0.3% Yeast extract, THBY for short, (Oxoid CM0189) and liquid culture was incubated for 24 h at $37\,^{\circ }\hbox {C}$ in the atmosphere with 5% CO2.

#### Simulation: diffusion-limited growth (dendritic patterns)

**Figure 4:**
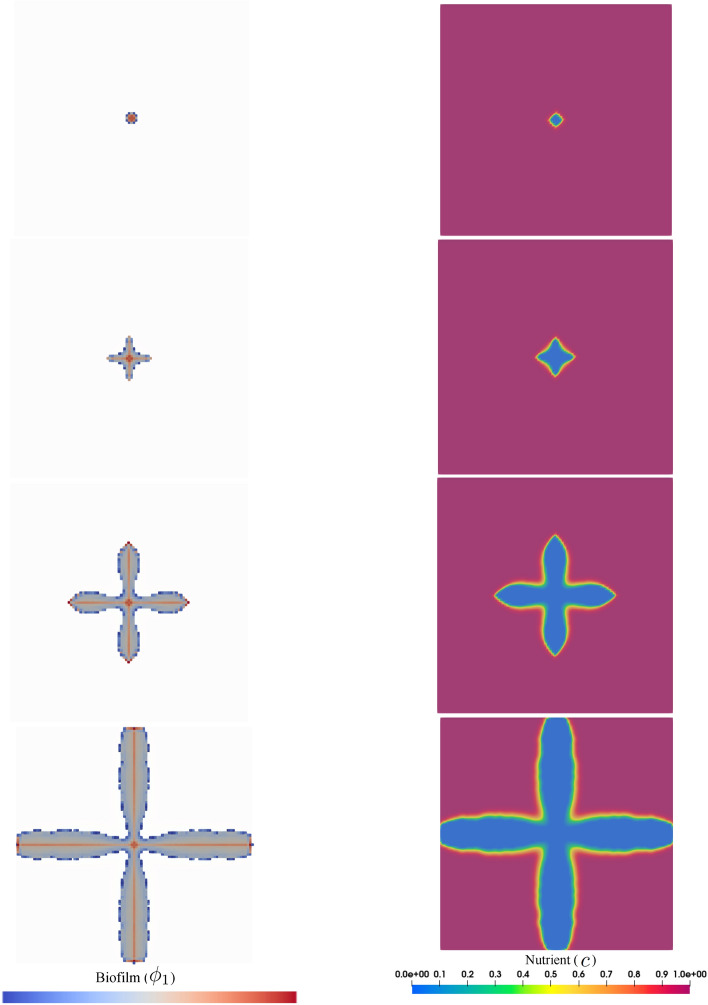
Diffusion-limited growth for $r_D=10^2$ (dendritic pattern) in the course of the time, from top to bottom.

If $r_D$ is relatively small, the biofilm tends to branch and form a dendritic shape. This is referred to as diffusion-limited growth in the literature. The formation of a finger-like structure, from a mathematical point of view, is rooted in the fluctuation-induced interface instability, see^39^. Such patterns are also observed in other phenomena such as multiphase flows^40^ and solidification process^41^. The formation of all irregular morphology with lobate margins can be explained in the context of nutrient absorption. It means that as a result of the scarcity of nutrient flux due to low diffusion through the biofilm, the bacteria increase the absorption surface via forming lobes. One can see that the nutrient is predominantly consumed at the locations where the biofilm exists and the far-field is not influenced by the growth. This is due to relatively low nutrient diffusivity that can not compensate for the consumed material. Looking at the pattern of nutrient consumption, one can realize that the nutrient is only depleted at the regions where biofilm exists. Figure 4 shows the numerical simulation of this scenario that is in qualitatively good agreement with the experimental observations reflected in Fig. 3 part b.

#### Simulation: diffusion-unlimited growth (compact pattern)

**Figure 5:**
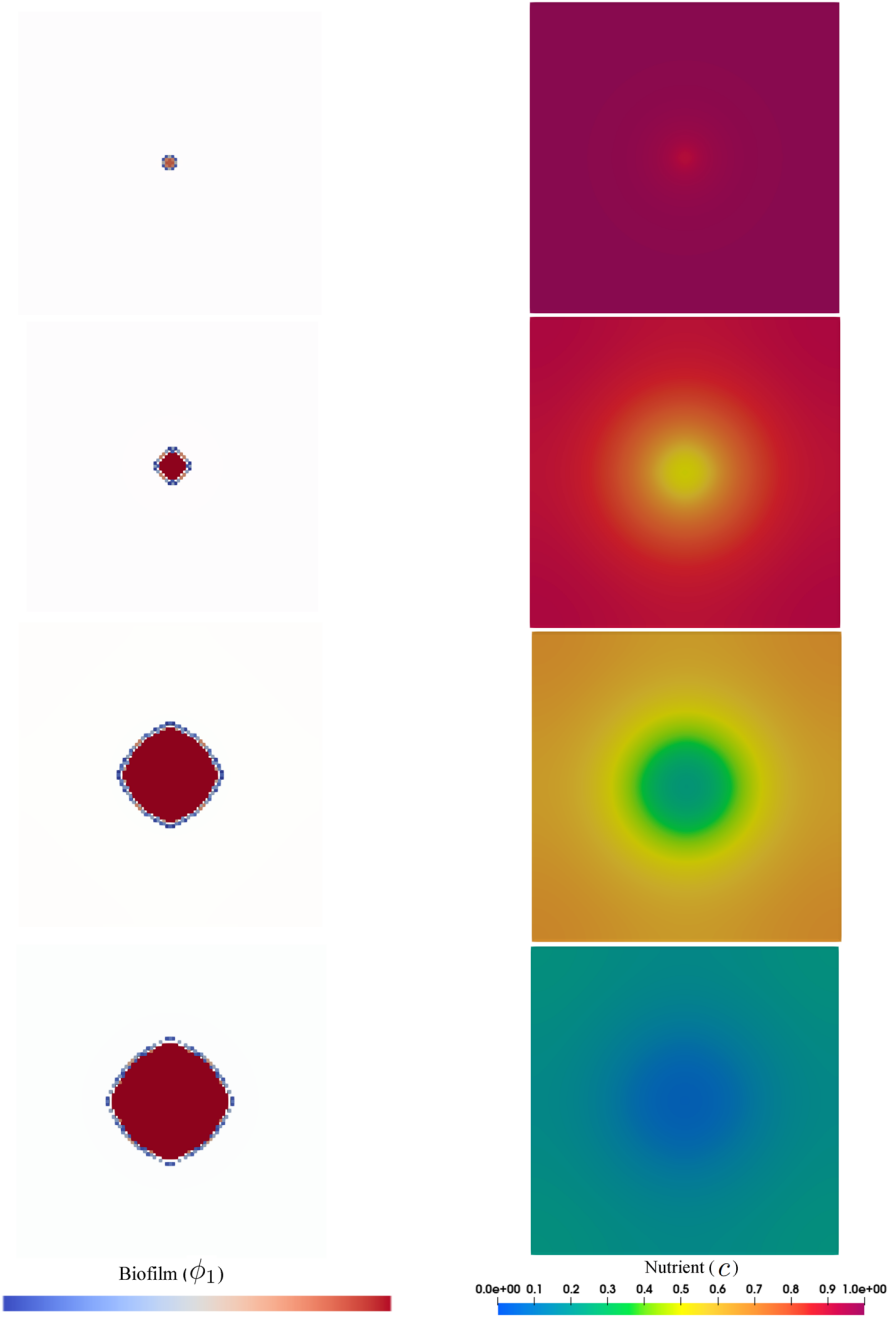
Diffusion-unlimited growth for $r_D=10^6$ (compact pattern) in the course of the time, from top to bottom.

If the diffusion of the nutrient is fast, the biofilm grows in a compact and spherical fashion, see Fig. 5. Unlike the previous example, the fast diffusion of nutrients ensures optimal supplies for the bacteria. Hence, the overall shape of the bacterial colony remains circular with entire margins. Here, due to the high diffusivity, the nutrient is transported from the whole domain to the biofilm-colonized regions. This leads to the gradual depletion of the nutrient in the whole domain, unlike the previous case. Experimental investigations also comply qualitatively with the mathematical model, see Fig. 3 part a.

### Test case 2: multiple colonies, two species, with interaction (co-aggregation)

#### Experiments: sample preparation for co-aggregation and biofilm formation

**Figure 6:**
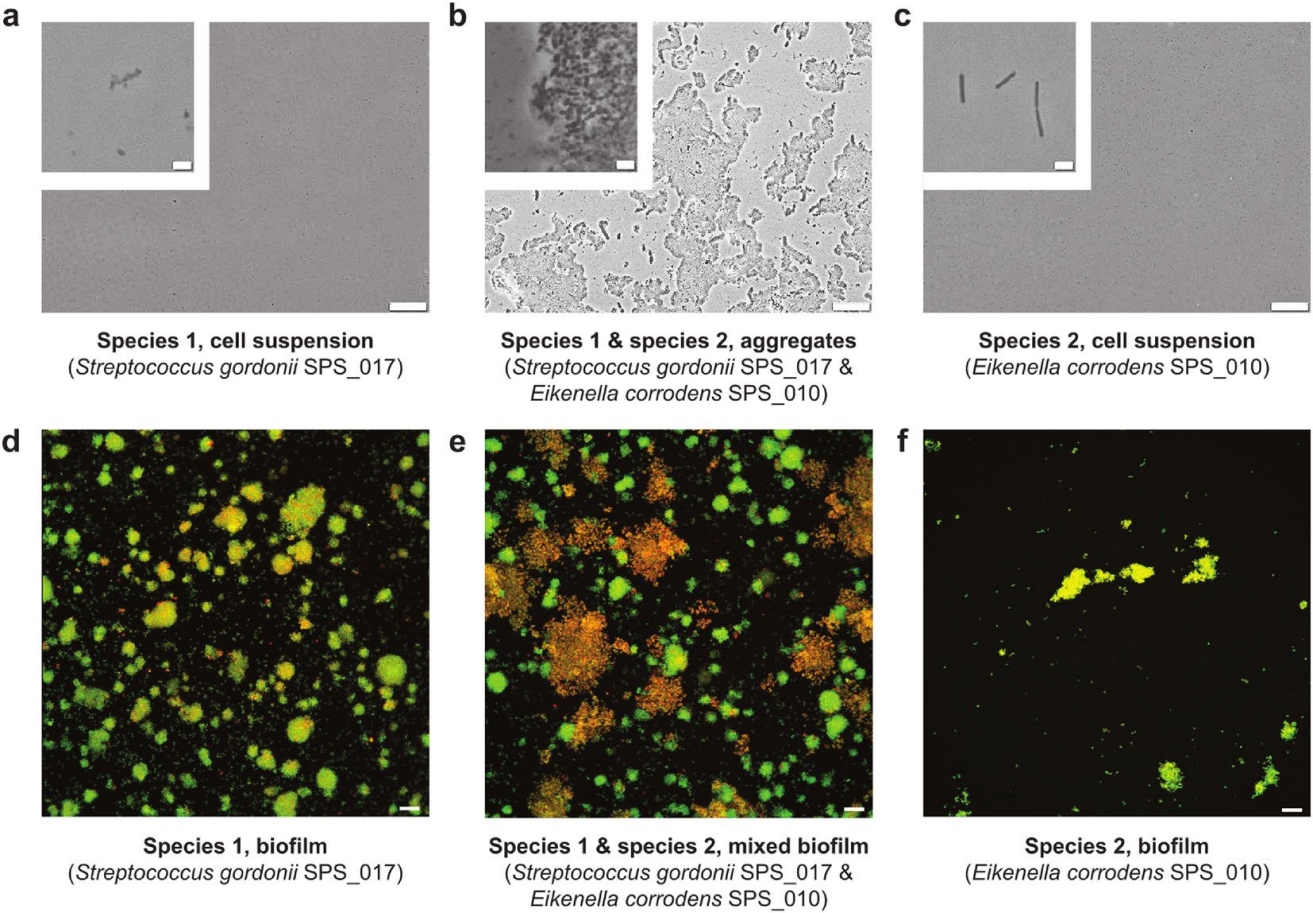
Co-aggregates and biofilms. a–c Low magnification phase-contrast micrographs of cell suspensions with a magnified subsection showing bacterial cells. From left: Streptococcus gordonii strain $SPS\_017$, S. gordonii strain $SPS\_017$ mixed with Eikenella corrodens strain $SPS\_010$, and E. corrodens strain $SPS\_010$. Both strains originate from the same severe case of peri-implant disease. After 24 h of incubation large co-aggregates were observed in the mixture but not in monospecies suspensions. Image fragments at high magnification are included in the top left corners. d–e Confocal micrographs of 8-h biofilms representing monoculture or co-culture biofilms are shown in the same order as in a–c. Bigger aggregates were observed in mixed-biofilms compared to monospecies biofilms. Scale bars, $4\,\upmu \hbox {m}$ (a–c, small bar), $10\,\upmu \hbox {m}$ (a-c, large bar), and $15\,\upmu \hbox {m}$ (d–e).

The bacteria were transferred to a co-aggregation buffer (1 mM Tris, 150 mM NaCl, 0.1 mM CaCl2, 0.1 mM MgCl2) and the optical density at 600 nm was set to 1.0. That is equal to a cell density of about 109 cells per ml. The co-culture inoculum was generated by mixing the same volumes of two species and resulted in the formation of co-aggregates. Monoculture and co-culture inocula were vortexed for 10 seconds, diluted ten times in co-aggregation buffer followed by mixing again. Then 3 ml of suspension was placed in 6-well plates (Thermo Fischer Greiner Bio-one Polystyrene 6-well Multiplates) and allowed to coat for 1 hour. Following this, the non-attached bacteria were removed from the wells and 3 ml of THBY medium was added. The bacteria were allowed to grow for 8 hours from this time point. After 8 hours of growth biofilms were stained with the LIVE/DEAD BacLight Bacterial Viability Kit (Invitrogen); rinsed, fixed in 2.5% glutaraldehyde solution in phosphate-buffered saline, and examined using CLSM (Leica TCS SP2, Leica Microsystems, Mannheim, Germany). For the imaging purpose, 488 nm laser was used with an emission range of 500 - 545 nm for SYTO9 and 590 - 680 nm for propidium iodide (PI). For the biofilm in each well, five representative images were acquired with optical sections of $3 \,\upmu \hbox {m}$. The Imaris Cell Imaging Software package (Imaris x64, 6.2.1, Bitplane AG, Zürich, Switzerland) was used to analyze images. A three-dimensional reconstruction smart view was generated, with green (SYTO9), red (PI) and yellow (co-localized SYTO9 and PI) fractions. Non-permeable cells were stained in green. Yellow and red signals were considered to represent permeable cells since PI penetrated the membrane of these cells. The experiment was performed twice. Each time for each group a total of two wells and ten images were generated. The experimental observations are illustrated in Fig. 6.

#### Simulation

In this test case, unlike test case 1, multiple colonies of two species are considered. The co-aggregation mechanism also exists and contributes to growth boosting. In order to replicate numerically the experimental observations shown in Fig. 6, the entire computational domain is randomly filled with initial colonies of both species in a uniform way. Then, we let the system evolve in time. The formation of the bacterial cluster in the regions with high proximity of the two species is observed as a result of co-aggregation.

**Figure 7:**
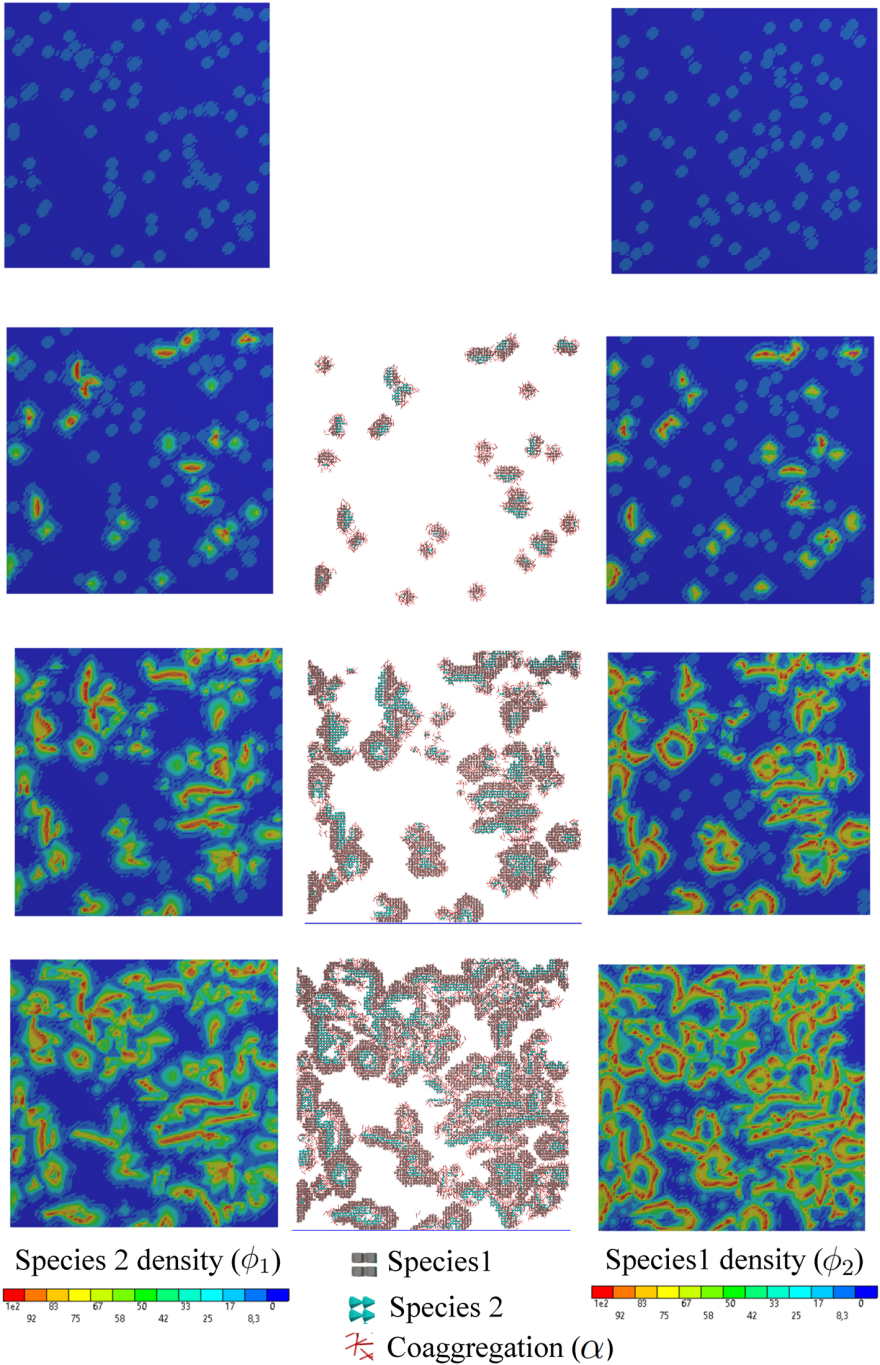
Diffusion-limited growth for $r_D=10^2$ in the course of the time, from top to bottom (see Supplementary Video 1).

**Figure 8:**
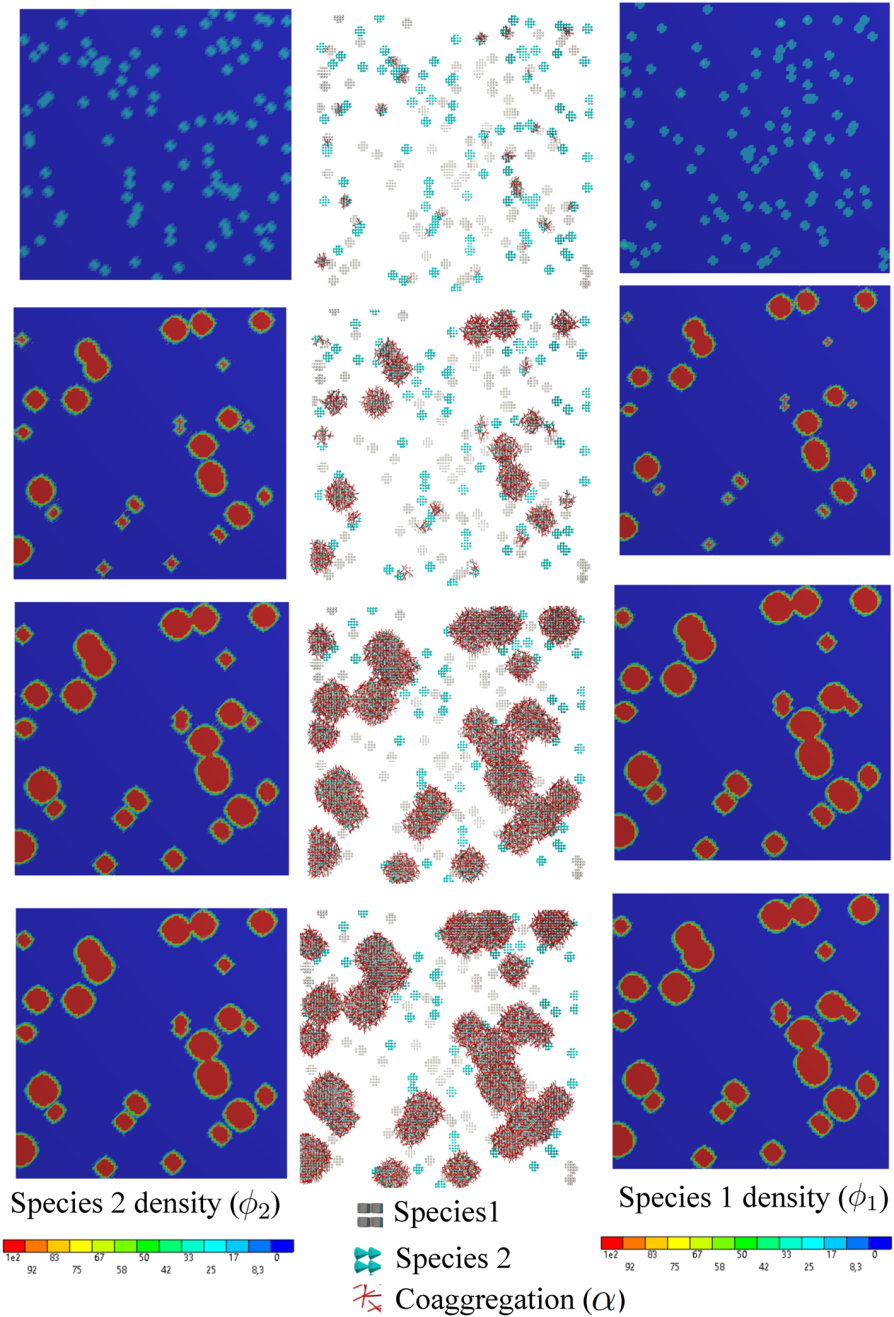
Diffusion-unlimited growth for $r_D=10^6$ in the course of the time, from top to bottom (see Supplementary Video 2).

Figures 7 and 8 show snapshots of both species being represented by tiny colonies with different colors (gray and cyan represent species 1 and species 2, respectively). Additionally, the regions where co-aggregation has taken place are pictured using the red rods. These two figures show the development of the colonies over the course of time for two different conditions discussed in the previous example, namely diffusion-limited and -unlimited growth. Similar to the previous test case, the appearance of the dendritic structure is the result of a diffusion-limited scenario (small $r_D$), while round and compact islands of colonies are a sign of diffusion-unlimited growth. These figures also demonstrate the nutrient distribution in the course of time. One can see that the nutrient consumption map is significantly different in the two cases. All in all, the numerical predictions show plausible qualitative compliance with the experimental observations.

**Figure 9:**
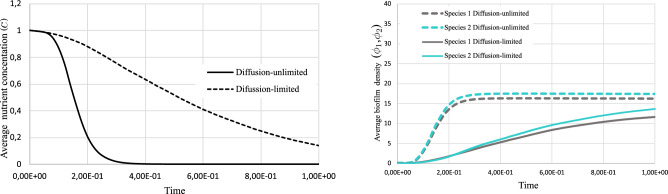
Multispecies biofilm growth under different nutrient diffusivity.

The average nutrient consumption is reflected in Fig. 9. While the nutrient concentration drops gradually in the diffusion-limited case, it is rapidly depleted in the diffusion-unlimited condition. In both cases, the profile of the average nutrient is monotonically decreasing because the uniform initial concentration is only consumed by the biofilm. It is also reflected in the average density of the biofilm that monotonically increases in both cases, however with different rates.

**Figure 10:**
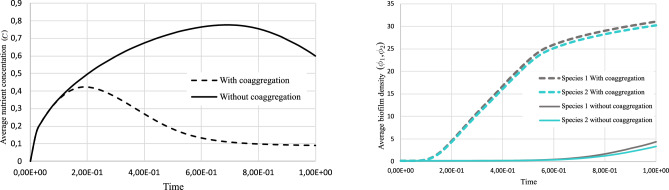
Multispecies biofilm growth under the directional nutrient provision for $r_D=10^6$.

### Test case 3: growth under the directional provision of nutrient with/without reciprocal interaction (co-aggregation)

This example is identical to the previous one with the difference that the nutrient is supplied constantly from a particular location (the top side, here). It leads to a prescribed gradient profile of the nutrient and consequently a preferred direction of colony expansion. To quantify the growth process, one can look at the average density of the biomass produced in time. It reflects how the available empty space is occupied by the two-species biofilm.

The experimental setup for such a test case is interesting and quite elaborate because the continuous and purely diffusion-driven feed of an aqueous environment from a specific location (one side) needs special care and measures. Any perturbation in the solution fluid can result in convective movement and mixing phenomenon which destroys the intended gradient in the nutrient availability. Unlike the experimental setting, the numerical modeling of the problem is free of such practical complications. One needs to apply the desired boundary condition on the intended regions. Hence, the experimental investigation is ruled out for this test case.

**Figure 11:**
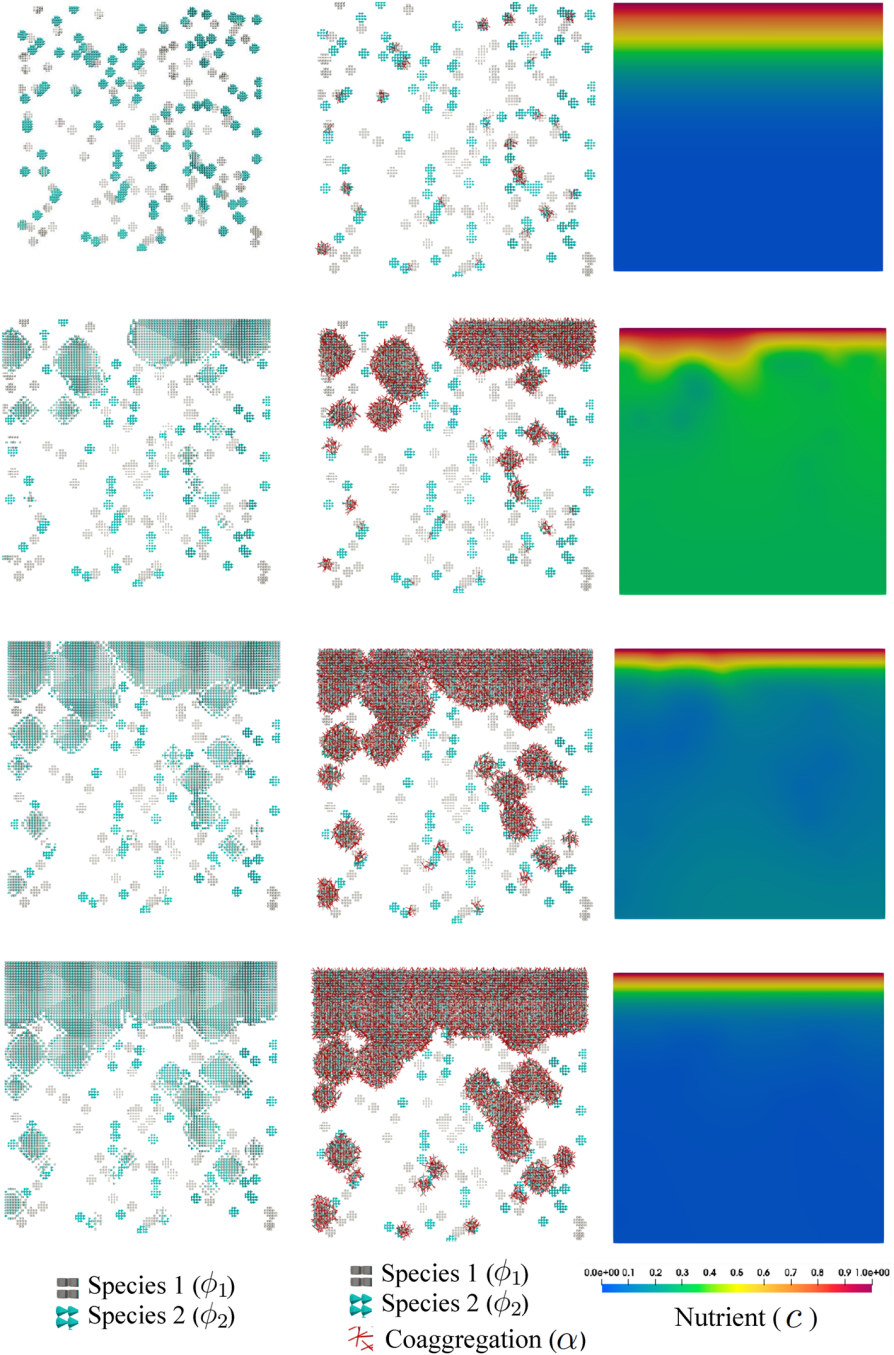
Directional nutrient feeding from the top side in the presence of co-aggregation for $r_D=10^6$ in the course of the time, from top to bottom (see Supplementary Video 3).

**Figure 12:**
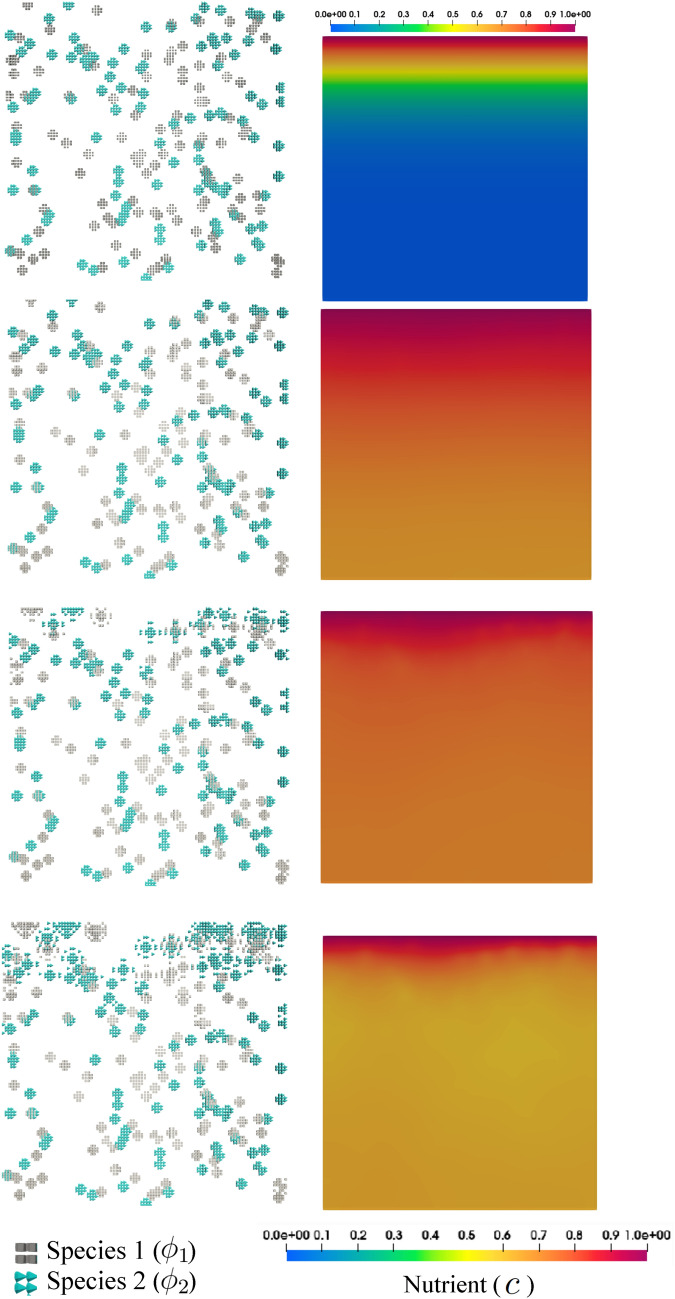
Directional nutrient feeding in the absence of co-aggregation for $r_D=10^6$ in the course of the time, from top to bottom.

The numerical simulation shows how the nutrient gradient affects the growth pattern of the biofilm. In fact, the final morphology is a result of both co-aggregation and nutrient availability. The biological activity of the bacteria is intensified according to the availability of co-aggregation partners as well as nutrient. Figs. 11 and 12 demonstrate snapshots of bacterial development in a dual-species system. The regions in which both species are coexistent are the dominant sites of bacterial growth which contribute to transforming tiny colonies into larger structured clusters of bacteria (biofilm). The clusters tend to form in the regions with high amounts of nutrients, namely the regions in the proximity of feeding sites at the top. Apart from nutrient feed, comparing Figs. 11 and 12 reveals how substantially the co-aggregation contributes to the rapid biofilm development.

In Fig. 10, the profile of the average nutrient and biofilm density is plotted with respect to the time. Due to the continuous provision of the nutrient and tiny initial colonies, the average value of the nutrient concentration increases first (primary ascending phase) and then decreases to a steady state constant value (secondary descending phase). The steady-state condition is reached when the rate of nutrient feeding is balanced with the rate of nutrient consumption when sufficient biofilm is developed. In the absence of co-aggregation, the development of biofilm, which determines the consumption capacity, is significantly slower. Consequently, the nutrient feed outweighs the nutrient consumption leading to an increase in the average nutrient and a delayed succeeding decrease.

## Conclusion

A novel mathematical model based on the phase-field method is proposed for the simulation of multi-species biofilms. The co-aggregation of the species is taken into account in a system of multi-species colonies. The complex morphology of multi-species colonies observed in the experiments can be reproduced using the presented mathematical model. The formation of such complex structures is driven, on one hand, by “nutrient availability” and, on the other hand, by “species proximity”. Since the proposed model is based on the phase-field approach, one does not need to track the moving interfaces of the multi-species and multi-colony biofilms. Handling multiple and moving boundaries is the main superiority of phase-field modeling in this application. Additionally, the merging of multiple colonies is treated naturally. It turns out that phase-field modeling is a successful and applicable approach for this biological problem at hand.

The nature of biofilm and the underlying processes are so complex that probably four continuous isotropic scalar variables ($\phi _1,\ \phi _2,\ c$ and $\alpha$) are not sufficient to capture the real bacterial morphology to a good extent. This is why quantitative validation of the numerical results against the experimental observations in this work is a challenging task. Nevertheless, mathematical modeling, despite simplifications, can help us to understand the “fundamental underlying mechanisms” that are involved.

This work can be extended and improved in several directions. A system composed of more than two species is of great interest and can be modeled just by adding phase-field equations. Furthermore, the interaction between the species can be generalized to not only reciprocal dual interaction but also to multilateral metabolic communications. Incorporating anisotropic growth function can also enhance the model. Finally, an efficient parallelized numerical architecture (such as MPI) can be employed to solve the model in 3D. These extensions are left for future work.

## Supplementary Information

Supplementary Legends.

Supplementary Video 1.

Supplementary Video 2.

Supplementary Video 3.

## Data Availability

Raw data (both numerical and experimental results) are available from the corresponding author on request. Furthermore, it is deposited into the Leibniz University public repository.

## Code availability

The developed FORTRAN file of the utilized ANSYS user element is available from the corresponding author on request. Furthermore, it is deposited into the Leibniz University public repository.

## References

[1] Kolenbrander PE, Palmer RJ, Periasamy S, Jakubovics NS (2010). Oral multispecies biofilm development and the key role of cell–cell distance. Nat. Rev. Microbiol..

[2] Kolenbrander PE, Palmer RJ, Rickard AH, Jakubovics NS, Chalmers NI, Diaz PI (2006). Bacterial interactions and successions during plaque development. Periodontology 2000.

[3] Mutha NVR, Mohammed WK, Krasnogor N, Tan GYA, Wee WY, Li Y, Choo SW, Jakubovics NS (2019). Transcriptional profiling of coaggregation interactions between streptococcus gordonii and veillonella parvula by dual RNA-seq. Sci. Rep..

[4] Palmer RJ, Shah N, Valm A, Paster B, Dewhirst F, Inui T, Cisar JO (2017). Interbacterial adhesion networks within early oral biofilms of single human hosts. Appl. Environ. Microbiol..

[5] Ding AM, Palmer RJ, Cisar JO, Kolenbrander PE (2010). Shear-enhanced oral microbial adhesion. Appl. Environ. Microbiol..

[6] Zhou P, Liu J, Merritt J, Qi F (2015). A YadA-like autotransporter, hag1 in Veillonella atypicais a multivalent hemagglutinin involved in adherence to oral streptococci, porphyromonas gingivalis, and human oral buccal cells. Mol. Oral Microbiol..

[7] Egland PG, Palmer RJ, Kolenbrander PE (2004). Interspecies communication in streptococcus gordonii–Veillonella atypica biofilms: Signaling in flow conditions requires juxtaposition. Proc. Natl. Acad. Sci..

[8] Kolenbrander PE (2011). Multispecies communities: interspecies interactions influence growth on saliva as sole nutritional source. Int. J. Oral Sci..

[9] Wu C, Chen Y-W, Scheible M, Chang C, Wittchen M, Lee JH, Luong TT, Tiner BL, Tauch A, Das A, Ton-That H (2021). Genetic and molecular determinants of polymicrobial interactions in fusobacterium nucleatum. Proc. Natl. Acad. Sci..

[10] Simon-Soro A, Ren Z, Krom BP, Hoogenkamp MA, Cabello-Yeves PJ, Daniel SG, Bittinger K, Tomas I, Koo H, Mira A (2022). Polymicrobial aggregates in human saliva build the oral biofilm. mBio.

[11] Kolenbrander PE (2000). Oral microbial communities: Biofilms, interactions, and genetic systems. Annu. Rev. Microbiol..

[12] Li Q, Wang H, Tan L, Zhang S, Lin L, Tang X, Pan Y (2021). Oral pathogen fusobacterium nucleatum coaggregates with pseudomonas aeruginosa to modulate the inflammatory cytotoxicity of pulmonary epithelial cells. Front. Cell. Infect. Microbiol..

[13] Lima BP, Hu LI, Vreeman GW, Weibel DB, Lux R (2018). The oral bacterium fusobacterium nucleatum binds staphylococcus aureus and alters expression of the staphylococcal accessory regulator sarA. Microb. Ecol..

[14] Lang C, Böttner M, Holz C, Veen M, Ryser M, Reindl A, Pompejus M, Tanzer J (2009). Specific lactobacillus/mutans streptococcus co-aggregation. J. Dent. Res..

[15] Lardon LA, Merkey BV, Martins S, Dötsch A, Picioreanu C, Kreft J-U, Smets BF (2011). iDynoMiCS: Next-generation individual-based modelling of biofilms. Environ. Microbiol..

[16] Naylor J, Fellermann H, Ding Y, Mohammed WK, Jakubovics NS, Mukherjee J, Biggs CA, Wright PC, Krasnogor N (2017). Simbiotics: A multiscale integrative platform for 3D modeling of bacterial populations. ACS Synth. Biol..

[17] Li B, Taniguchi D, Gedara JP, Gogulancea V, Gonzalez-Cabaleiro R, Chen J, McGough AS, Ofiteru ID, Curtis TP, Zuliani P (2019). NUFEB: A massively parallel simulator for individual-based modelling of microbial communities. PLoS Comput. Biol..

[18] Tack ILMM, Nimmegeers P, Akkermans S, Hashem I, Impe JFMV (2017). Simulation of escherichia coli dynamics in biofilms and submerged colonies with an individual-based model including metabolic network information. Front. Microbiol..

[19] Verhulst A, Cappuyns A, Derlinden EV, Bernaerts K, Impe JV (2011). Analysis of the lag phase to exponential growth transition by incorporating inoculum characteristics. Food Microbiol..

[20] Alpkvista E, Klapper I (2007). A multidimensional multispecies continuum model for heterogeneous biofilm development. Bull. Math. Biol..

[21] Albero AB, Ehret AE, Böl M (2014). A new approach to the simulation of microbial biofilms by a theory of fluid-like pressure-restricted finite growth. Comput. Methods Appl. Mech. Eng..

[22] Soleimani M (2019). Finite strain visco-elastic growth driven by nutrient diffusion: theory, FEM implementation and an application to the biofilm growth. Comput. Mech..

[23] Jana S, Charlton SGV, Eland LE, Burgess JG, Wipat A, Curtis TP, Chen J (2020). Nonlinear rheological characteristics of single species bacterial biofilms. npj Biofilms Microbiomes.

[24] Helmlinger G, Netti PA, Lichtenbeld HC, Melder RJ, Jain RK (1997). Solid stress inhibits the growth of multicellular tumor spheroids. Nat. Biotechnol..

[25] Cyron CJ, Wilson JS, Humphrey JD (2014). Mechanobiological stability: A new paradigm to understand the enlargement of aneurysms?. J. R. Soc. Interface.

[26] Golding I, Kozlovsky Y, Cohen I, Ben-Jacob E (1998). Studies of bacterial branching growth using reaction–diffusion models for colonial development. Physica A.

[27] Shen Y, Zhao J, de la Fuente-Núñez C, Wang Z, Hancock REW, Roberts CR, Ma J, Li J, Haapasalo M, Wang Q (2016). Experimental and theoretical investigation of multispecies oral biofilm resistance to chlorhexidine treatment. Sci. Rep..

[28] Stewart PS, Zhang T, Xu R, Pitts B, Walters MC, Roe F, Kikhney J, Moter A (2016). Reaction-diffusion theory explains hypoxia and heterogeneous growth within microbial biofilms associated with chronic infections. npj Biofilms Microbiomes.

[29] Wood BD, Whitaker S (1998). Diffusion and reaction in biofilms. Chem. Eng. Sci..

[30] Wood BD, Whitaker S (2000). Multi-species diffusion and reaction in biofilms and cellular media. Chem. Eng. Sci..

[31] Fisher RA (1937). the wave of advance of advantageous genes. Annals Eugenics.

[32] Kot, M. *Elements of Mathematical Ecology* (Cambridge University Press, 2001).

[33] Rickard AH, Gilbert P, High NJ, Kolenbrander PE, Handley PS (2003). Bacterial coaggregation: An integral process in the development of multi-species biofilms. Trends Microbiol..

[34] Cuny L, Pfaff D, Luther J, Ranzinger F, Ödman P, Gescher J, Guthausen G, Horn H, Hille-Reichel A (2019). Evaluation of productive biofilms for continuous lactic acid production. Biotechnol. Bioeng..

[35] Feng G, Cheng Y, Wang S-Y, Borca-Tasciuc DA, Worobo RW, Moraru CI (2015). Bacterial attachment and biofilm formation on surfaces are reduced by small-diameter nanoscale pores: How small is small enough?. npj Biofilms Microbiomes.

[36] Allen SM, Cahn JW (1979). A microscopic theory for antiphase boundary motion and its application to antiphase domain coarsening. Acta Metall..

[37] Steinbach, I. & Salama, H. *Lectures on Phase Field*. Springer Nature Switzerland, (2023).

[38] Korelc, J. & Wriggers, P. *Automation of Finite Element Methods* (Springer International Publishing, 2016).

[39] Kessler DA, Levine H (1998). Fluctuation-induced diffusive instabilities. Nature.

[40] Saffman PG (1986). Viscous fingering in Hele-Shaw cells. J. Fluid Mech..

[41] Kupferman R, Shochet O, Ben-Jacob E (1994). Numerical study of a morphology diagram in the large undercooling limit using a phase-field model. Phys. Rev. E.

