# Supplementary material for: Numerical and experimental investigation of multi-species bacterial co-aggregation
Source: Sci Rep. 2023 Jul 22;13:11839. doi: 10.1038/s41598-023-38806-2 (PMC10363141; doi:10.1038/s41598-023-38806-2)
Supplement: Supplementary file 1 — Supplementary Legends. [file 41598_2023_38806_MOESM1_ESM.docx]

**Supplementary Video Legends**

**Supplementary Video 1:** Diffusion-limited growth for $r_{D}={10}^{2}$in the course of the time (See Figure 11 for more details)

**Supplementary Video 2:** Diffusion-unlimited growth for $r_{D}={10}^{6}$in in the course of the time (See Figure 8 for more details)

**Supplementary Video 3:** Directional nutrient feeding from the top side in the presence of co-aggregation for $r_{D}={10}^{6}$in the course of the time (See Figure 7 for more details)
